# Compromised Anti-inflammatory Action of Neutrophil Extracellular Traps in PAD4-Deficient Mice Contributes to Aggravated Acute Inflammation After Myocardial Infarction

**DOI:** 10.3389/fimmu.2019.02313

**Published:** 2019-10-01

**Authors:** Kaveh Eghbalzadeh, Leena Georgi, Theresa Louis, Haizhi Zhao, Ugur Keser, Carolyn Weber, Martin Mollenhauer, Andreas Conforti, Thorsten Wahlers, Adnana Paunel-Görgülü

**Affiliations:** ^1^Department of Cardiothoracic Surgery, Heart Center, University of Cologne, Cologne, Germany; ^2^Department of Cardiology, Heart Center, University of Cologne, Cologne, Germany; ^3^Center for Molecular Medicine Cologne, University of Cologne, Cologne, Germany

**Keywords:** neutrophil extracellular traps, inflammation, PAD4, myocardial infarction, macrophages

## Abstract

Innate immune responses and rapid recruitment of leukocytes, which regulate inflammation and subsequent healing, play a key role in acute myocardial infarction (MI). Peptidylarginine deiminase 4 (PAD4) is critically involved in chromatin decondensation during the release of Neutrophil Extracellular Traps (NETs) by activated neutrophils. Alternatively, activated macrophages (M2) and accurate collagen deposition determine the repair of the infarcted heart. In this study, we investigated the impact of NETs on macrophage polarization and their role for acute cardiac inflammation and subsequent cardiac healing in a mouse model of acute MI. NETs were found to promote *in vitro* macrophage polarization toward a reparative phenotype. NETs suppressed pro-inflammatory macrophages (M1) under hypoxia and diminished IL-6 and TNF-α expression. Further on, NETs strongly supported M2b polarization and IL-10 expression. In cardiac fibroblasts, NETs increased *TGF-ß* expression under hypoxic culture conditions. PAD4^−/−^ mice subjected to permanent ligation of the left anterior descending artery suffered from overwhelming inflammation in the acute phase of MI. Noteworthy, PAD4^−/−^ neutrophils were unable to release NETs upon *ex vivo* stimulation with ionomycin or PMA, but produced significantly higher amounts of reactive oxygen species (ROS). Increased levels of circulating cell-free DNA, mitochondrial DNA and cardiac troponin were found in PAD4^−/−^ mice in the acute phase of MI when compared to WT mice. Reduced cardiac expression of *IL-6, IL-10*, and M2 marker genes, as well as increased *TNF-*α expression, suggested a pro-inflammatory state. PAD4^−/−^ mice displayed significantly increased cardiac *MMP-2* expression under baseline conditions. At day 1, post-MI, PAD4^−/−^ mice showed increased end-diastolic volume and increased thinning of the left ventricular wall. Interestingly, improved cardiac function, as demonstrated by significantly increased ejection fraction, was found at day 21. Altogether, our results indicate that NETs support macrophage polarization toward an M2 phenotype, thus displaying anti-inflammatory properties. PAD4 deficiency aggravates acute inflammation and increases tissue damage post-MI, partially due to the lack of NETs.

## Introduction

Myocardial infarction (MI) leads to the death of up to one billion cardiac cells in response to occurred ischemia and, in consequence, the affected myocardium undergoes a series of remodeling processes accompanied by inflammatory responses, fibrosis and finally scar formation (1). Infarct healing and subsequent remodeling might lead to impaired cardiac function resulting in chronic heart failure, a major cause of high morbidity and mortality (2). Thus, healing of the infarcted heart substantially affects patients' outcome and, therefore, regulation of this process might offer a potential therapeutic strategy for patients suffering from MI. During the first hours after MI, neutrophils massively infiltrate into the infarct area and release cytokines and chemokines, as well as reactive oxygen species (ROS), which exacerbate vascular and tissue injury. Moreover, activated neutrophils release decondensated chromatin covered by elastase, myeloperoxidase (MPO) and cytoplasmic proteins, known as neutrophil extracellular traps (NETs). This process of NETs formation is called NETosis and largely differs from apoptosis and necrosis. Although NETs may contribute to the clearance of bacteria during infection, they are additionally involved in many pathological conditions, such as deep vein thrombosis (3), ischemia/reperfusion (4), atherosclerosis (5), cancer and trauma (6, 7), amongst others. Formation of NETs strongly depends on the enzyme peptidylarginine deiminase 4 (PAD4), which citrullinates arginine residues on histone tails resulting in chromatin decondensation. Accordingly, PAD4-deficient mice were shown to lack NETosis yielding in significant protection from myocardial ischemia reperfusion injury (4). Similarly, increased neutrophil counts are prognostic for larger infarct size (8) and depletion of neutrophils in a canine ischemic reperfusion model was shown to reduce infarct size (9). A recent study has demonstrated that, beside neutrophils, monocytes become rapidly recruited after ischemia onset (10). Monocytes/macrophages exhibit high phenotypic and functional plasticity and several macrophage phenotypes have already been described. At early times with a peak around day 3, macrophages are polarized to pro-inflammatory M1 macrophages, whereas over time, a shift to alternatively activated M2 cells occurs, which promote resolution of inflammation and tissue remodeling (11). M2 macrophages were subdivided into M2a, M2b, and M2c cells according to their functional properties and activation mechanisms. M2a and M2c cells are crucial for promoting the adaptive immune response and clearance of apoptotic cells (12), whereas, M2b cells are largely involved in the suppression and regulation of inflammation by the secretion of high levels of IL-10 (13). Impaired resolution of inflammation and prolonged M1 macrophage activation may aid adverse cardiac remodeling and impair MI outcomes. In this regard, silencing of interferon regulatory factor 5 (IRF5), which regulates polarization toward the M1 phenotype, shifted macrophage phenotype from M1 to M2 in the heart and attenuated heart failure post-MI (14).

It is widely known that fibroblasts are critically involved in the reparative response following MI and are implicated in the pathogenesis of cardiac remodeling (15). Injury induces fibroblast proliferation, migration to the injured areas and transdifferentiation into myofibroblasts, which express contractile proteins such as α- smooth muscle actin and might contribute to cardiac fibrosis. Myofibroblasts produce large amounts of collagens, which are crucial for preventing rupture of the ventricular wall. Whereas, ROS and transforming growth factor-ß (TGF-ß) signaling have been reported to play a major role in fibroblast activation, recently. Fibroblasts were demonstrated to undergo transdifferentiating to collagen-producing myofibroblasts after incubation with NETs *in vitro*, and NETs were found in proximity to α-smooth muscle actin (α-SMA)-positive fibroblasts in tissue sections from patients with fibrotic intestinal lung disease (16). Hence, pro-fibrotic signals secreted at the infarction site might be involved in the development of reactive fibrosis in the non-infarcted myocardium (17). Whether NETs contribute to adverse remodeling has not been investigated so far. Further on, although activated neutrophils and macrophages interact closely in the infarcted heart, nothing is known about the effect of NETs regarding macrophage polarization and, thus, about their immunoregulatory properties. In the present study, we therefore aimed to explore the effects of NETs on macrophage polarization and acute inflammation and to further prove their effects on cardiac regeneration post-MI using an established acute MI model.

## Materials and Methods

### Animals

PAD4-deficient mice were generated by mating *Pad4*^*flox*/*flox*^ with *Tg(CMV-Cre)1Cgn* mice to delete exons 9–10. All mice were obtained from the Jackson laboratory. C57BL/6J wildtype mice served as controls. Mice were housed at 22–24°C in a 12/12 h light/dark cycle and given free access to water and standard rodent chow. Experiments on mice were performed at 9–12 weeks of age. All animal studies were reviewed and approved by the local animal care committee (Landesamtes für Natur, Umwelt und Verbraucherschutz (LANUV), Germany, No.84-02.04.2014.A234) and were in accordance with the Guide for the care and Use of Laboratory Animals.

### Induction of MI

MI was induced by permanent ligation of the left anterior descending coronary artery (LAD). Before surgery, buprenorphine (0.1 mg/kg) was administered s.c. Male WT and PAD4^−/−^ were anesthetized with 2% isoflurane, then intubated and ventilated with a standard rodent ventilator (MiniVent Ventilator for Mice, Harvard Apparatus). The thoracic cavity was opened and the LAD was ligated with 8/0 polypropylene suture placed through the myocardium into the anterolateral LV wall. Sham operated animals were subjected to thoracotomy without LAD ligation. Infarction was confirmed by echocardiography and Evans blue staining of excised hearts.

### Quantification of Infarct Size

Mice were sacrificed at the end of experiment and hearts were excised and cut into four 2-mm sliced. Each section was incubated in 1% 2,3,5-triphenyltetrazolium chloride (TTC, Sigma-Aldrich) solution for 15 min at 37°C. Both sides of each TTC-stained tissue slice were photographed using a digital camera. Infarct size is expressed as a percentage of total left ventricular mass.

### Echocardiographic Analyses

Transthoracic echocardiography was performed with a Vevo 3100 (VisualSonics, Inc., Toronto, ON, Canada) system. Mice were maintained under anesthesia with continuously delivered 2% isoflurane gas inhalation and additional s.c. buprenorphine application (0.1 mg/kg). Examination was conducted on a temperature-controlled platform to prevent hypothermia caused by the ultrasound gel and to ensure physiological heart and respiratory rates. Ultrasound gel was applied to the depilated skin of the chest. Imaging was obtained with a MX550d transducer (40 MHz center transmit, axial resolution 40 μm; VisualSonics, Inc.). Baseline cardiac function was determined in a cohort of healthy non-operated WT and PAD4^−/−^ mice. Sham operated and permanent LAD ligation cohorts of WT and PAD4^−/−^ mice both underwent echocardiographic measurements at days 1, 3, and 21 post-MI. Comprehensive left ventricular function was analyzed as already published elsewhere (18).

### Isolation and Polarization of Murine Macrophages

Bone marrow cells were isolated by flushing femurs of 9–12 weeks old C57/Bl6 mice. Cells (1.7–2 × 10^6^) were cultured in 6-well plates in RPMI medium supplemented with 20% FCS containing 20 ng/ml recombinant murine M-CSF (Peprotech), 100 U/ml penicillin and 10 μg/ml streptomycin (Sigma Aldrich) in a humidified incubator at 37°C. On days 3 and 6, the medium was changed. Differentiated macrophages (M0) were obtained after 7 days of culture.

For *in vitro* polarization, M0 macrophages were cultured in RPMI medium with 10% FCS supplemented with 20 ng/ml recombinant murine IFN-ɤ (Peprotech) and 100 ng/ml LPS (Sigma Aldrich) to induce a M1-like phenotype or in medium supplemented with 20 ng/ml recombinant murine IL-4 (Peprotech) to induce M2a-like macrophages, respectively.

### Isolation of Cardiac Fibroblasts

Cardiac fibroblasts were isolated from excised hearts of 8–12 weeks old C57/Bl6J wildtype mice. In brief, heart tissue was cut into small pieces and digested in collagenase II (100 U/ml) and trypsin (0.2%) in HBSS at 37°C for 30 min using C Tubes (Miltenyi), and following dissociation by gentle MACS Dissociator (Miltenyi). The digested tissue was filtered through a 70 μm cell strainer and cells were centrifuged at 300 × *g* for 5 min and washed in PBS. Cells were seeded in T75 flasks and the medium was carefully replaced after 2 h. Cells were cultured in DMEM/F12 medium supplemented with 10% FCS at 37°C and 5% CO_2_ and split at a confluency of ~80%. In this study, cardiac fibroblasts at passage 3 have been used.

### Isolation of Murine Bone-Marrow Neutrophils

Neutrophils were isolated from the bone marrow of wildtype C57/Bl6J and PAD4^−/−^ mice. Mice were euthanized, femurs and tibias were flushed with HBSS + 3% FCS and cells were passed through a 70 μm strainer to remove tissue. Red blood cells were hypotonically lysed using 0.2% NaCl, followed by the addition of 1.2% NaCl to stop lysing. Neutrophils were then isolated by density gradient centrifugation using 62% percoll (GE Healthcare). Cells were gently laid on percoll and centrifuged at 1,000 × *g* for 30 min at 15°C without break. Neutrophils were found in the pellet and washed twice with HBSS + 3% FCS.

### Cell Culture Conditions

For cell culture experiments, cells were cultured under normoxia (21% O_2_, 5% CO_2_) at 37°C for 20 h. In parallel experiments, cells were placed in a hypoxia chamber (Stem cell technologies) and cultured under hypoxic conditions (2% O_2_, 5% CO_2_) at 37°C for equivalent periods of time.

### Flow Cytometry

Hearts excised at day 1 post-MI were digested with collagenase II (450 U/ml), hyaluronidase type I-S (60 U/ml) and DNase (60 U/ml) in DMEM, and filtered through a 40 μm cell strainer. After lysis of red blood cells (Multi-species RB Lysis Buffer, eBioscience), immune cells were identified by CD45 staining (FITC rat anti-mouse, clone 30-F11, BD). Dead cells were excluded by propidium iodide staining (1 μg/ml) and live singlets were gated by FSC-A/FSC-H. To identify macrophages/monocytes, cells were additionally stained using F4/80 APC (clone BM8, Bio Legend) antibodies and samples were quantified by flow cytometry using MACS Quant Analyzer (Miltenyi Biotec). The percentage of CD45^+^/F4/80^+^ cells was calculated from the total number of vital cells.

### *In vitro* Neutrophil Stimulation and Generation of NETs-Enriched Supernatants

To induce NETs release, bone marrow-derived neutrophils (4 × 10^6^) were seeded into 6-well culture plates and stimulated with 100 nM phorbol myristate acetate (PMA; Sigma Aldrich) for 3.5 h. In some experiments cells were stimulated with 4 μM ionomycin (Cayman Chemicals). For NETs isolation, supernatants were carefully discarded and cells were washed once with PBS. PBS was then added and residual neutrophils and NETs were collected by vigorous pipetting. Cell debris were centrifuged at 50 × *g* for 5 min at 4°C and NETs-enriched supernatants were collected and stored at −80°C until use. DNA/NETs concentration was quantified using a NanoDrop spectrophotometer (Thermo Fisher) at a wavelength of 260 nm.

### Determination of Peroxidase Activity in NETs-Enriched Supernatants

Peroxidase activity in supernatants from simulated neutrophils was measured by mixing a 50 μl sample with 50 μl detection solution (1:1 TMB Substrate A/TMB Substrate B, BioLegend) in a 96-well plate. After 3 min incubation at room temperature, the staining reaction was stopped by adding 50 μl H_2_SO_4_. The absorbance was measured at 450 nm using a microplate reader (Victor X3, Perkin Elmer).

### Quantification of ROS Production

To quantify ROS production in neutrophils, freshly isolated neutrophils were suspended in RPMI + 2% FCS + 2 mM Ca^2+^ and 3–4 × 10^5^ cells (200 μl) were added into 96 well plates and stimulated with different concentrations of ionomycin and PMA for 3 h at 37°C. Then, 20 μM DHR-123 (PromoKine) was added to the wells and plates were incubated under shaking for 15 min at 37°C. Plates were placed on ice and further centrifuged at 1,500 rpm for 5 min at 4°C. Supernatants were removed and cells were washed with PBS. Finally, cells were suspended in 200 μl PBS and DHR-123 conversion into the fluorophore rhodamine-123 (R-123) was detected at 485 nm using Victor X3 microplate reader (Perkin Elmer).

### Purification of cfDNA From Plasma

cfDNA was isolated from plasma samples of WT and PAD4-deficient mice at day 1 after MI induction by the method previously described (19).

### NETs Induction and Immunofluorescence Staining

To induce NETosis, freshly isolated bone marrow-derived neutrophils (1.5 × 10^5^) were seeded on poly-L-lysine coated coverslips. Cells were stimulated with 4 μM ionomycin for 3 h. After that, cells were fixed with 4% PFA, blocked and incubated with a polyclonal rabbit anti-myeloperoxidase antibody (1:100, Abcam), or alternatively, with a rabbit anti-histone H3 (citrulline R2+R8+R17) antibody (1:500, Abcam). Cells were further incubated with a secondary goat anti-rabbit Alexa Fluor 488-conjugated antibody (1:1,000, Cell Signaling Technology), counterstained with DAPI and mounted in Dako fluorescent mounting medium (Dako). NETs were finally visualized using an inverted microscope (Eclipse Ti-U 100, Nikon) and the NIS Elements BR 3.10 software package.

### Quantification of NETs and Plasma cfDNA

Freshly isolated neutrophils were suspended in RPMI + 2% FCS+ 2 mM Ca^2+^ and 2 × 10^5^ cells (200 μl) were added into 96 well plates and stimulated with 4 μM ionomycin for 3 h at 37°C. Pico Green (Invitrogen) was then added to the wells and plates were incubated for 3 min in the dark. After plate centrifugation at 50 × *g* for 5 min, 200 μl supernatant was carefully transferred into a new plate. Fluorescence intensity was measured in a multiplate reader (Victor X3, Perkin Elmer) at excitation and emission wavelengths of 485 nm and 530 nm. Culture medium without cells was used as blank control.

cfDNA levels in plasma samples were quantified by Pico Green staining as previously described (19).

### Immunoblotting

Cell lysates were loaded onto acrylamide gels and transferred to nitrocellulose membrane. Membranes were blocked and incubated with the following antibodies according to manufacturer's protocols: polyclonal rabbit anti-mouse Collagen I α 1, rabbit anti-mouse Collagen III α 1 (both Novus Biologicals) and polyclonal rabbit anti-mouse ß-Actin, rabbit anti-mouse Smad3, and rabbit monoclonal anti-mouse Phospho-Smad3 (Ser 423/425, clone C25A9) (Cell Signaling Technology).

Membranes were then exposed to anti-rabbit horseradish peroxidase as secondary antibody (Dako), and signal detection was performed using ECL detection reagent (Thermo Fisher).

### Real Time PCR

Total RNA was extracted using RNeasy Mini Kit (Qiagen) or Tri Reagent (Sigma Aldrich) according to the manufacturer's instructions. Contaminating DNA was removed by DNA-*free* Kit DNA Removal Kit (Ambion). RNA was reverse transcribed using High Capacity cDNA Reverse Transcription Kit (Applied Biosystems). For Real time PCR, gene-specific primers for *iNOS* (20), *Arg I* (21), *TNF-*α (22), *IL-10* (23), *TGF-ß* (24), *MMP-2* (25), *MMP-9* (26), *Collagen-1, Collagen-3* (27), *IL-6, 18S RNA* (19)*, Fizz1/RELM*α, *Ym-1* (28), *SPHK1, LIGHT* (29), *MertK* (30), were used. All samples were run in triplicates. Relative gene expression levels were determined using PowerUP SYBR Green PCR Master Mix (Applied Biosystems) according to the manufacturer's recommended protocol with the following thermal cycling conditions: 2 min 50°C, 2 min 95°C, 40 cycles of 1 s 95°C and 30 s 60°C, and 4°C hold (QuantStudio 3 Real-Time PCR System, Applied Biosystems). Expression of target genes was normalized to the endogenous control *18S* RNA gene. Fold expression was calculated using the 2^−ΔΔ*Ct*^ methods. In some experiments, the 2^Δ*Ct*^ method was used to determine relative gene expression.

### Mitochondrial DNA Quantification

The content of mtDNA in plasma was assessed using a specific primer for mouse mtDNA and nuclear B2m (31). Real time PCR was performed using 5 ng of template DNA and 800 nM of each primer. All samples were analyzed in duplicate. The average threshold cycle number (Ct) values were determined in the same quantitative PCR run. The level of mtDNA was calculated relative to nuclear B2m as follows: relative copy number 2^Δ*Ct*^, whereby ΔCt = Ctnuc – Ctmit.

### ELISAs

For cytokine quantification in culture supernatants, the following ELISA kits have been used: Human/mouse TGF beta 1 2nd Generation ELISA Ready-SET-Go (eBioscience), Mouse IL-6 DuoSet ELISA (R&D Systems), Mouse IL-10 DuoSet ELISA (R&D Systems) and Mouse TNF alpha ELISA Ready-SET-Go (eBioscience) following the manufacturers' instructions. Cardiac troponin (cTnT) was quantified using Mouse cTnT/TNNT2 ELISA Kit (Immunoway).

A mouse multiplex Immunoassay (ProcartaPlex 4 Plex, Thermo Fisher) and Luminex 200 System (Thermo Fisher) were used to quantify cytokines in plasma samples.

### Statistical Analyses

Data were analyzed with GraphPad Prism 5 software. Experimental data are presented as means with standard error of the mean (SEM). Datasets were assessed for normality using the Kolmogorov-Smirnov test. Unpaired data of two groups were analyzed using unpaired *t*-test. Normally distributed unpaired data of multiple groups were analyzed using one-way ANOVA with Newman Keuls *post-hoc* test. In cases of non-normal distribution, Mann-Whitney was used. *P*-value less that 0.05 was considered as statistically significant.

## Results

### NETs Alter Macrophage Phenotype and Enhance IL-10 Expression *in vitro*

**Figure 1:**
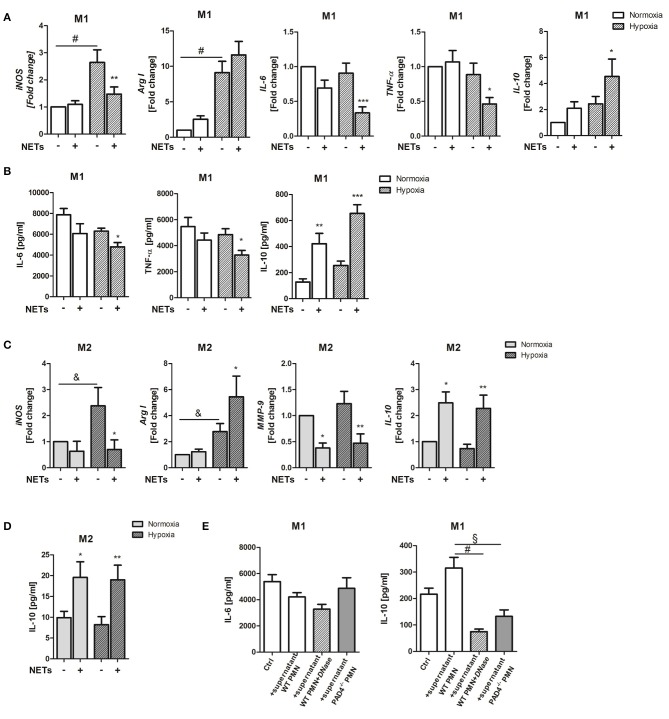
Effects of NETs on macrophage phenotype and inflammatory markers *in vitro*. **(A)** Macrophages (M0) were cultured in the presence of M1-polarizing cytokines and NETs-enriched supernatants (1,000 ng/ml) under normoxia or hypoxia for 24 h. Gene expression of M1-/M2-related makers (*iNOS, Arg I, IL-6, TNF-*α, *IL-10*) was analyzed by Real time PCR. *n* = 10. **(B)** The expression of IL-6, TNF-α, and IL-10 in culture supernatants was quantified by ELISA. *n* = 4–6. **(C)** In parallel experiments, macrophages were cultured in the presence of M2a-polarizing cytokines and NETs. Gene expression of *iNOS, Arg I, MMP-9*, and *IL-10* was examined by Real time PCR. *n* = 10. **(D)** IL-10 in culture supernatants was quantified by ELISA. *n* = 6. **(E)** In some control experiments, macrophages were cultured in the presence of M1-polarizing cytokines and normoxic conditions and further stimulated with NETs-enriched supernatant from PMA-stimulated WT neutrophils (1,000 ng/ml), with NETs pre-digested by *DNase I* and with supernatant of stimulated PAD4^−/−^ neutrophils, respectively. After 24 h, the concentration of secreted IL-6 and IL-10 was quantified by ELISA. *n* = 6. ^*^p < 0.05; ^**^p < 0.01; ^***^p < 0.001 vs. control sample under same culture conditions. ^&^*p* < 0.05; ^§^*p* < 0.01; ^#^*p* < 0.001. Two-way ANOVA was performed followed by Newman-Keuls *post-hoc* test.

Cardiac healing following MI involves inflammation and tissue remodeling. Macrophage polarization has recently been recognized to define resolution of inflammation and the quality of cardiac healing (32). NETs have for a long time been considered to possess pro-inflammatory actions. We, therefore, investigated the impact of NETs for the inflammatory response to MI in respect to their ability to influence macrophage phenotype *in vitro*. To this end, we used NETs-enriched supernatants from *in vitro* activated bone marrow-derived neutrophils. Initial experiments performed to estimate the proper concentration of NETs demonstrated that, at a concentration of 1,000 ng/ml, NETs induce significant differences between control and NETs-treated group. Bone marrow-derived macrophages from WT mice were stimulated with 1,000 ng/ml NETs under M1- (IFN-ɤ + LPS) or M2a (IL-4)-polarizing culture conditions. To imitate ischemia in the infarcted heart, cells were cultured under hypoxic condition (2% O_2_) and cells cultured under normoxia (21% O_2_) served as a control. In the presence of IFN-ɤ and LPS and hypoxic conditions, NETs significantly suppressed *inducible nitric oxide synthase* (*iNOS), IL-6* and *TNF-*α gene expression and slightly upregulated *Arg I* expression, which is a marker of the M2 phenotype (Figure 1A). Of note, *IL-10* expression was significantly upregulated. Accordingly, reduced IL-6 and TNF-α levels and increased IL-10 secretion could be observed (Figure 1B).

Under M2a-polarizing conditions, NETs suppressed *iNOS* and *matrix metalloproteinase (MMP)-9* expression (Figure 1C). MMP-9 has been reported to be upregulated in macrophages after IL-4 stimulation (33). In turn, upregulation of *arginase I* (*Arg I)* and *IL-10* gene and IL-10 protein expression were detected (Figures 1C,D). Although NETs exerted more pronounced effects under hypoxia, IL-10 was also strongly regulated under normoxic conditions, indicating that NETs might modulate macrophage IL-10 expression in the non-ischemic myocardium.

To verify if IL-10 indeed becomes regulated by NETs, in some experiments, macrophages cultured under normoxic conditions were treated with NETs pre-digested with *DNase I* and supernatant collected from ionomycin-stimulated PAD4^−/−^ neutrophils, respectively, which were shown to be unable to release NETs (34). As shown in Figure 1E, NETs-mediated effects could not be reproduced with digested DNA or DNA released by PAD4^−/−^ cells, due to other cell death mechanisms which differ from NETosis. These results suggest that NETs release post-MI may drive macrophage polarization toward an anti-inflammatory phenotype.

**Figure 2:**
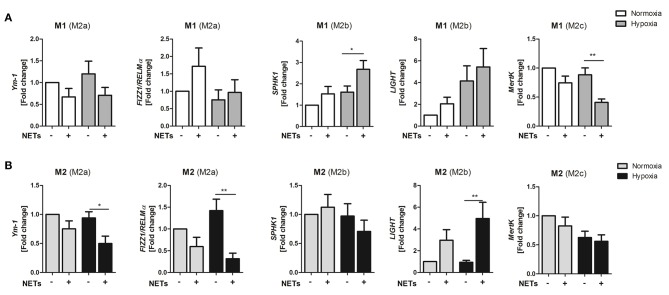
NETs promote upregulation of M2b marker genes *in vitro*. Macrophages (M0) were cultured in the presence of M1- **(A)** or M2a- **(B)** polarizing cytokines and 1,000 ng/ml NETs under normoxia or hypoxia for 24 h. Marker genes specific for the M2a (*Ym1, Fizz1*/*RELM*α), M2b (*SPHK1, LIGHT*), and M2c (*MertK*) subtypes were analyzed by Real time PCR. *n* = 8–10. ^*^p < 0.05; ^**^p < 0.01. Two-way ANOVA was performed followed by Newman-Keuls *post-hoc* test.

Because NETs obviously promote M2 polarization under pro- and anti-inflammatory culture conditions, we next investigated if NETs favor polarization toward one of the three macrophage subpopulations, termed M2a, M2b, and M2c. Treatment with NETs under M1-polarizing conditions and hypoxia upregulated the M2b marker *SPHK1* but suppressed the M2c marker *MertK* (Figure 2A). Similarly, we also observed upregulation of the M2b marker *LIGHT* under M2a-polarizing conditions. In addition, downregulation of the M2a marker genes, *Ym-1*, and *Fizz/RELM-*α, could be detected, indicating suppression of wound healing and fibrosis markers (Figure 2B).

The backbone of NETs mainly consists of chromatin, which in turn includes histone proteins, DNA and nucleosomes. All of these components have been described to exert different immunomodulatory effects (35). To find out whether gene regulation at least partially depends on NETs-triggered pathways, we next performed Toll-like receptor 9 (TLR-9) blocking experiments. As NETs were found to strongly suppress *IL-6* and to increase *IL-10* expression under M1-polarizing conditions, as well as to strongly upregulate *IL-10* and *LIGHT* under M2a-polarizing conditions (Figures 1, 2), we chose these genes to elaborate whether TLR-9 is involved in NETs-induced signaling pathways. Inhibition of endosomal acidification by chloroquine did not influence NETs-mediated down-regulation of *IL-6* expression, suggesting that this cytokine is not regulated by DNA, but rather by other NETs components. On the contrary, no upregulation of *IL-10* under M1-polarizing conditions, as well as *IL-10* and *LIGHT* under M2a-polarizing conditions, could be observed after blockage of TLR-9 (Supplementary Figure 1). Similar results were obtained when IL-10 was detected in culture supernatants (data not shown). Thus, regulation of *IL-10* and *LIGHT* expression by NETs occurs via TLR-9 signaling.

### NETs Influence the Activity of Cardiac Fibroblasts *in vitro*

**Figure 3:**
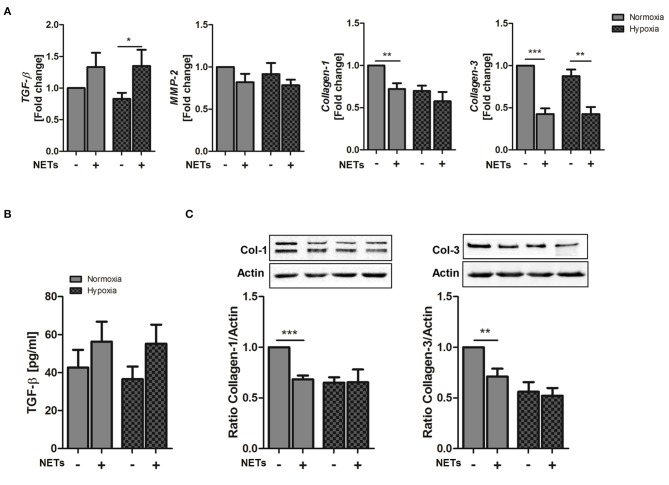
Effect of NETs on collagen expression in cardiac fibroblasts *in vitro*. Cardiac fibroblasts were stimulated with 1,000 ng/ml NETs and cultured under normoxic and hypoxic conditions for 24 h. **(A)** Expression of *TGF-ß, MMP-2, Collagen-1*, and *Collagen-3* was evaluated by Real time PCR. *n* = 10. **(B)** TGF-ß levels were quantified in culture supernatants. *n* = 6. **(C)** The expression of Collagen-1 and Collagen-3 in cardiac fibroblasts was analyzed by Western blot. *n* = 65−7. ^*^p < 0.05; ^**^p < 0.01; ^***^p < 0.001. Two-way ANOVA was performed followed by Newman-Keuls *post-hoc* test.

Cardiac fibroblasts are critically involved in both reparative and fibrotic processes and their activity determines cardiac healing and remodeling in distinct ways. Excessive proliferation and profibrotic activity might favor adverse remodeling and cardiac fibrosis (36). To determine whether NETs regulate fibrosis-related factors, we additionally treated cardiac fibroblasts with NETs under hypoxia and normoxia (Figure 3). NETs significantly increased *TGF-ß* mRNA expression in cardiac fibroblasts cultured under hypoxic conditions. *MMP-2* expression, which is associated with adverse remodeling (37), did not change, whereas, significant *collagen-1* and *-3* suppression was found to occur after incubation with NETs under normoxic conditions. NETs also reduced *collagen-3*, but not *collagen-1*, expression under hypoxic conditions (Figure 3A). Although TGF-ß protein secretion by fibroblasts was found to be slightly upregulated by NETs (Figure 3B), we observed an inhibition of collagen-1 and−3 protein expression under normoxic, but not under hypoxic culture conditions (Figure 3C). No regulation of the α-SMA gene and protein expression was detected (data not shown).

### Neutrophils From PAD4-Deficient Mice Are Unable to Release NETs but Produce Higher Levels of ROS

**Figure 4:**
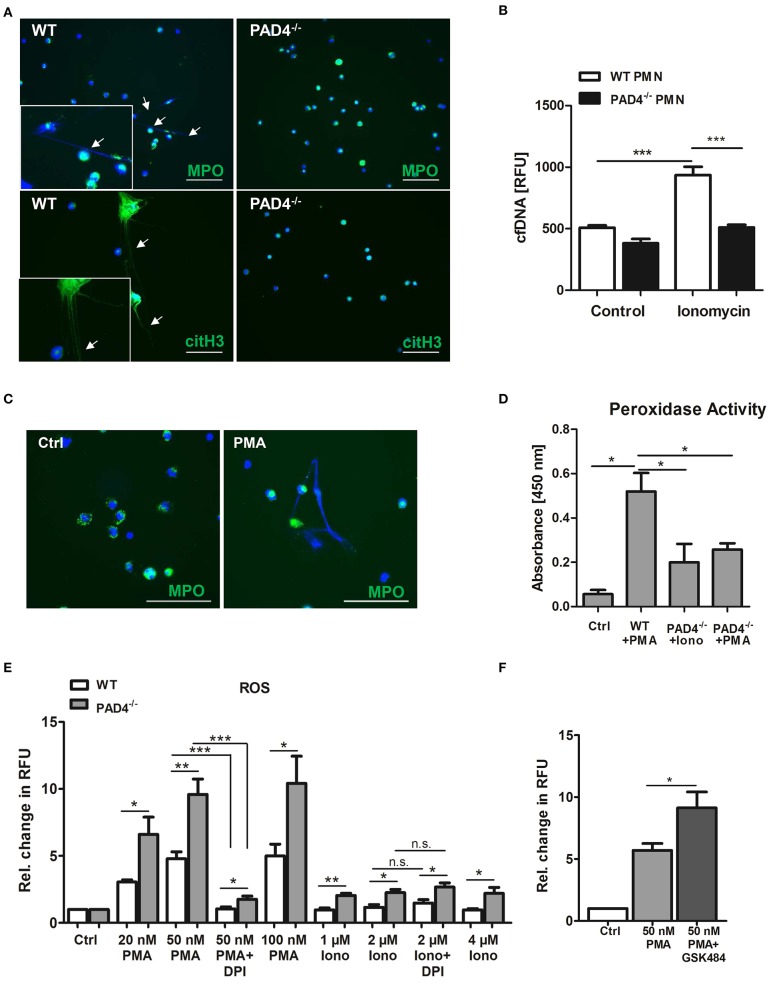
PAD4^−/−^ neutrophils are unable to release NETs but produce high amounts of ROS. **(A)** Bone marrow-derived neutrophils from WT and PAD4^−/−^ mice were seeded on coated coverslips and stimulated with 4 μM ionomycin for 3 h at 37°C. For NETs detection, cells were stained with anti-MPO antibody or anti-citrullinated histone H3 antibody (*both green*) and counterstained with DAPI (*blue*). Representative images of five independent experiments are depicted. Scale bar = 50 μm. **(B)** WT and PAD4^−/−^ neutrophils were stimulated with 4 μM ionomycin for 3 h and cfDNA/NETs release in culture supernatants was quantified by Pico Green DNA staining. *n* = 5. ^***^p < 0.001. Two-way ANOVA was performed followed by Newman-Keuls *post-hoc* test. **(C)** Bone marrow-derived WT neutrophils were seeded on coverslips and stimulated with 100 nM PMA for 3 h to release NETs. NETs were detected by staining with anti-MPO antibody (*green*) and DAPI (*blue*). Representative images of three independent experiments are shown. Arrow heads indicate NETs formation. Scale bar = 50 μm. **(D)** Peroxidase activity in supernatants collected from WT neutrophils stimulated with PMA (100 nM) and from PAD4^−/−^ neutrophils stimulated with PMA or ionomycin (4 μM), respectively, was quantified by the addition of TMB substrate and absorbance measurement. *n* = 3. ^*^p < 0.05. Two-way ANOVA was performed followed by Newman-Keuls *post-hoc* test. **(E)** Bone marrow-derived neutrophils (3 × 10^5^) from WT and PAD4^−/−^ mice were stimulated with the indicated concentrations of PMA and ionomycin for 3 h. In some experiments, cells were pre-incubated with DPI (10 μM) for 20 min. Then, DHR-123 (20 μM) was added and cells were incubated for additional 15 min at 37°C under shaking. Relative change in RFU compared to unstimulated control samples is depicted. *n* = 4. ^*^p < 0.05; ^**^p < 0.01; ^***^p < 0.001 determined by the Student *t*-test. **(F)** Bone marrow-derived WT neutrophils (4 × 10^5^) were pre-cultured in the presence of GSK484 for 20 min before stimulation with 50 nM PMA for 3 h. After addition of DHR-123, cells were further incubated for 15 min under shaking at 37°C. Fluorescence of Rhodamine-123 was measured with a microplate reader. Relative change in RFU is depicted. ^*^p < 0.05 determined by the Student *t*-test.

To test whether bone marrow-derived neutrophils from PAD4^−/−^ mice are able to release NETs we stimulated cells with the calcium ionophore ionomycin, which induces hyperactivation of PADs and protein citrullination. As depicted in Figure 4, only WT neutrophils formed NETs (Figure 4A) and released cfDNA in culture supernatants (Figure 4B) upon stimulation, whereas NETs formation was abrogated in the absence of functional PAD4. Similarly, stimulation of WT neutrophils with PMA provoked NETs release (Figure 4C), correlating with upregulated peroxidase activity in culture supernatants (Figure 4D). On the contrary, only diminished peroxidase activity was measured in supernatants of PAD4^−/−^ neutrophils incubated with ionomycin and PMA, respectively.

The production of ROS by NADPH oxidase is considered to be a hallmark in the process of NETosis. To find out whether PAD4 deficiency is attributed to altered oxidative burst in leukocytes, we stimulated WT and PAD4^−/−^ bone marrow-derived neutrophils with different concentrations of PMA and ionomycin and quantified ROS by DHR-123 staining. Of note, cells deficient for active PAD4 produced significantly higher amounts of ROS when compared to WT cells after stimulation with both PMA and ionomycin (Figure 4E). Because PMA is a strong activator of the NADPH oxidase complex, pre-incubation of cells with the NADPH oxidase inhibitor DPI strongly suppressed ROS production. In turn, ionomycin did not stimulate NADPH oxidase activity and no effect of DPI was found. Importantly, higher levels of ROS were found in PAD4^−/−^ neutrophils after ionomycin stimulation, suggesting increased NADPH oxidase-independent ROS production. Inhibition of PAD4 activity in WT neutrophils by GSK484 significantly increased ROS production upon PMA stimulation (Figure 4F). These results suggest a possible regulation of the NADPH oxidase complex and other intracellular ROS sources by PAD4.

### PAD4-Deficient Mice Display Higher Levels of cfDNA, cTnT and Pro-Inflammatory Mediators in the Acute Phase Post-MI

**Figure 5:**
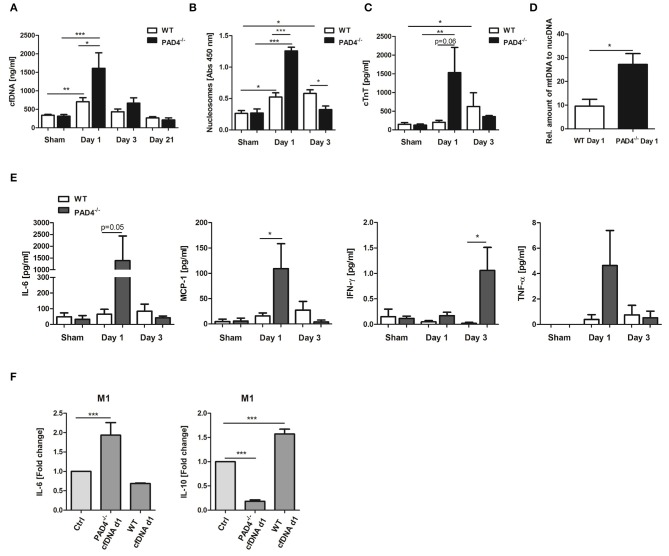
Impact of PAD4-deficiency on the plasma levels of cfDNA, nucleosomes, cardiac troponin and inflammatory markers after MI. cfDNA **(A)**, nucleosomes **(B)**, and cardiac troponin (cTnT) **(C)** levels were quantified in plasma samples of WT and PAD4^−/−^ mice at day 1, day 3, and day 21 after MI. *n* = 5–12. ^*^p < 0.05; ^**^p < 0.01; ^***^p < 0.001. Intra-group differences were determined by two-way ANOVA with Newman-Keuls *post-hoc* test. For inter-group differences, the Student *t*-test was used. **(D)** Relative quantification of plasma mtDNA at day 1 post-MI. *n* = 5. ^*^p < 0.05 determined by Student *t*-test. **(E)** Plasma levels of IL-6, MCP-1, IFN-ɤ, and TNF-α were quantified by ProcartaPlex Multiplex Assay. *n* = 5–6. ^*^p < 0.05 determined by the Mann-Whitney test. **(F)** Macrophages (M0) were cultured under M1-polarizing culture conditions and stimulated with cfDNA (600 ng/ml) isolated from plasma of WT and PAD4^−/−^ mice at day 1 post-MI. After 24 h, levels of IL-6, and IL-10 were quantified in culture supernatants by ELISA. Fold change in expression vs. unstimulated control cells is depicted. *n* = 6. ^***^p < 0.001 determined by two-way ANOVA followed by Newman-Keuls *post-hoc* test.

An increase in cfDNA after MI is widely believed to be a consequence of MI-associated cell death and neutrophil activation, which release NETs. Having shown that PAD4^−/−^ neutrophils do not release NETs *ex vivo*, we next tested if PAD4 deficiency results in diminished cfDNA elevation after MI in mice, and if so, whether these mice display more profound signs of inflammation. Although significant elevation in cfDNA (Figure 5A) and nucleosome levels (Figure 5B) were detected in WT mice at day 1 post-MI, surprisingly, this increase was more prominent in PAD4^−/−^ mice, and significant intergroup differences were found at that time. However, at day 3, nucleosome levels were significantly lower in PAD4^−/−^ mice compared to WT mice. To address whether enhanced cfDNA release in PAD4^−/−^ mice in the acute phase post-MI might be due to increased damage of the myocardium, cardiac troponin T (cTnT) levels in plasma were additionally quantified (Figure 5C). Indeed, in PAD4-deficient mice, cTnT amounts were already significantly elevated at day 1 post-MI and were somehow higher when compared to the amounts detected in WT mice. In contrast, cardiac damage in WT mice was delayed and cTnT levels increased first at day 3 post-MI. In line with this, significantly higher levels of circulating mtDNA were detected in PAD4^−/−^ mice at day 1 supporting the hypothesis that PAD4 deficiency aggravates tissue damage in the acute phase (Figure 5D). Accordingly, increased plasma levels of IL-6, monocyte chemoattractant protein-1 (MCP-1), and TNF-α were found at day 1 and of IFN-ɤ at day 3 post-MI in PAD4^−/−^ mice, respectively (Figure 5E). IL-10 levels remained below detection levels at all time points (not shown).

We next assessed whether circulating cfDNA from PAD4^−/−^ mice, obviously released by damaged cells, acts different to NETs. For this, macrophages were stimulated with 600 ng/ml DNA isolated from day 1 plasma of mice subjected to LAD ligation (Figure 5F). Plasma-derived cfDNA from PAD4^−/−^ mice strongly increased IL-6 secretion and inhibited IL-10 release by macrophages under M1-polarizing conditions, supporting the pro-inflammatory nature of cfDNA. Importantly, plasma-derived cfDNA from WT mice (day 1) was found to increase IL-10 expression in M1 cells, thus showing NETs-like properties.

### Different Cardiac Expression of Inflammation-Related Genes in PAD4-Deficient Mice at Day 1 Post-MI

**Figure 6:**
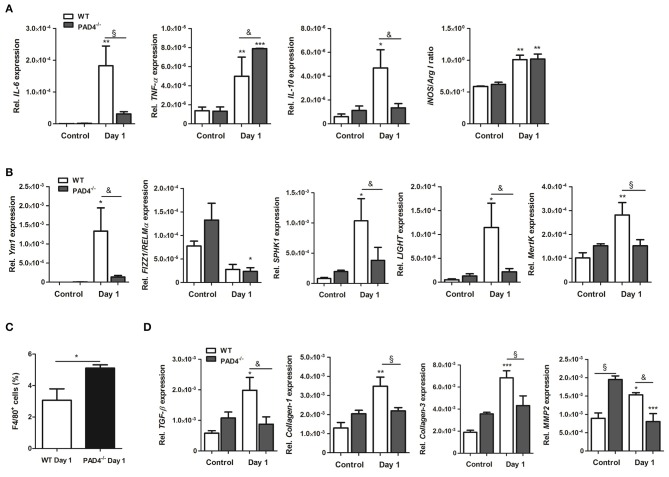
Cardiac expression of inflammation-related genes in PAD4^−/−^ mice at day 1 post-MI. **(A)** Cardiac gene expression of *IL-6, TNF-*α, *IL-10, iNOS*, and *Arg I* was determined by Real time PCR in mice subjected to permanent LAD ligation (*n* = 3–4) and control animals (*n* = 3–5). The ratio of *iNOS* to *Arg I* is depicted. **(B)** To examine polarization toward the M2 phenotype, marker genes specific for the M2a (*Ym1, Fizz1*/*RELM*α), M2b (*SPHK1, LIGHT*), and M2c (*MertK*) subtypes were analyzed by Real time PCR. *n* = 3–5. ^*^p < 0.05; ^**^p < 0.01; ^***^p < 0.001 vs. control. ^&^*p* < 0.05; ^§^*p* < 0.01. Two-way ANOVA was performed followed by Newman-Keuls *post-hoc* test. **(C)** Percentage of F4/80^+^ cells was quantified by flow cytometry in dissociated hearts from WT (*n* = 3) and PAD4^−/−^ (*n* = 4) mice. ^*^p < 0.05 determined by Student *t*-test. **(D)** Cardiac expression of *TGF-ß, Collagen-1, -3* and *MMP2* was assessed by Real time PCR. *n* = 3-5. ^*^p < 0.05; ^**^p < 0.01; ^***^p < 0.001 vs. control. ^&^*p* < 0.05; ^§^*p* < 0.01. Two-way ANOVA was performed followed by Newman-Keuls *post-hoc* test.

To further examine the inflammatory response within infarcted hearts, we quantified the expression of inflammatory genes and macrophage marker genes by Real time PCR. As displayed in Figure 6A, the expression of *IL-6* and *IL-10* significantly increased in the infarcted hearts of WT mice. In contrast, significantly higher levels of *TNF-*α were found in PAD4^−/−^ hearts at day 1 post-MI, whereby *IL-6* and *IL-10* expression were lower than in WT hearts. Both mouse strains showed a significantly higher ratio of *iNOS* to *Arg I*, confirming M1 polarization at day 1 post-MI. In addition, the M2a marker *Ym1*, the M2b markers *SPHK1*, and *LIGHT* and the M2c marker *MertK* markedly increased in infarcted hearts of WT, but not in PAD4^−/−^ mice (Figure 6B). In accordance with increased MCP-1 plasma levels found in PAD4^−/−^ mice (Figure 5E), FACS analyses revealed enhanced recruitment of F4/80^+^ cells in infarcted PAD4^−/−^ hearts (Figure 6C). Given that NETs also influenced the expression of collagens, the cardiac expression of *collagen-1* and *-3* was additionally quantified. Notably, the cardiac expression of both genes was significantly upregulated only in WT mice at day 1 post-MI but did not increase in PAD4^−/−^ hearts. Similarly, cardiac expression levels of *TGF-ß* and *MMP-2* were significantly reduced in PAD4-deficient mice when compared to WT mice (Figure 6D). Of note, control PAD4^−/−^ mice displayed increased cardiac *MMP-2* expression when compared to WT mice, suggesting that PAD4 deficiency does not solely influence the formation of NETs.

### Increased Acute Inflammation in PAD4-Deficient Mice Does Not Compromise Cardiac Healing and Function in the Post-Acute Phase of MI

**Figure 7:**
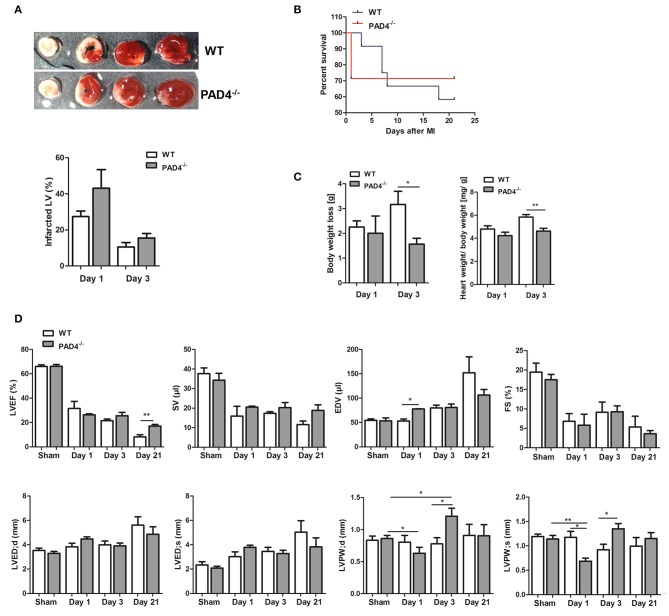
PAD4^−/−^ mice show better recovery at post-acute stages. **(A)** Quantification of infarct sizes at day 1 (*n* = 3) and day 3 (*n* = 5) post-MI. **(B)** Survival curves of WT and PAD4^−/−^ mice subjected to permanent LAD ligation for 21 days (*n* = 7–12). **(C)** Weight loss and heart to body weight ratio in WT and PAD4^−/−^ mice at day 1 and day 3 post-MI. *n* = 3–5. **(D)** LVEF, SV, EDV, and FS were determined at days 1 (*n* = 3), 3 (*n* = 8), and 21 (*n* = 4–5) post-MI by transthoracic echocardiography. Additionally, differences in cardiac dimensions (LVED;d, LVED;s, LVPW;d, LVPW;s) between mice were quantified. ^*^p < 0.05; ^**^p < 0.01 determined by Student *t*-test.

Excessive persistent inflammation was previously described to favor adverse remodeling (38). We therefore investigated whether overwhelming acute inflammation in PAD4^−/−^ mice negatively influences cardiac healing. Consistent with increased cTnT levels, we found slightly increased infarct size in the hearts of PAD4^−/−^ mice at day 1 when compared to those of WT mice, although not statistically significant (Figure 7A). Of note, although survival rate post-MI was not significantly influenced by the genotype, PAD4^−/−^ mice were found to solely die during the acute phase of MI (day 1) and all mice survived at later times (Figure 7B). Remarkably, PAD4^−/−^ mice that survived acute inflammation showed reduced body weight loss and significantly lower heart weight to body weight ratios at day 3 post-MI when compared to WT mice (Figure 7C). Echocardiographic analyses revealed increased end-diastolic volume (EDV) and increased thinning of the left ventricular posterior wall (LVPW) in PAD4^−/−^ mice at day 1, although the left ventricular ejection fraction (LVEF) was not reduced (Figure 7D). Surprisingly, the LVPW was significantly thicker in PAD4^−/−^ hearts already at day 3 post-MI and LVEF was significantly higher at day 21 post-MI. In addition, stroke volume (SV) was slightly increased and the end-diastolic volume (EDV), as well as the left ventricular end-diastolic diameter (LVED), tended to be lower when compared to WT mice, suggesting reduced cardiac hypertrophy and remodeling in this group.

## Discussion

Acute MI leads to the death of cardiomyocytes, which induces the release of DAMPs, including DNA (39), and a strong pro-inflammatory response, in order to remove the damaged tissue. Numerous previous studies reported that NETs released by activated neutrophils under pathological conditions possess pro-inflammatory deleterious effects and favor adverse cardiac events (4, 40–44). This study investigated the impact of NETs on macrophage polarization and their role in acute inflammation and cardiac healing post-MI.

**Figure 8:**
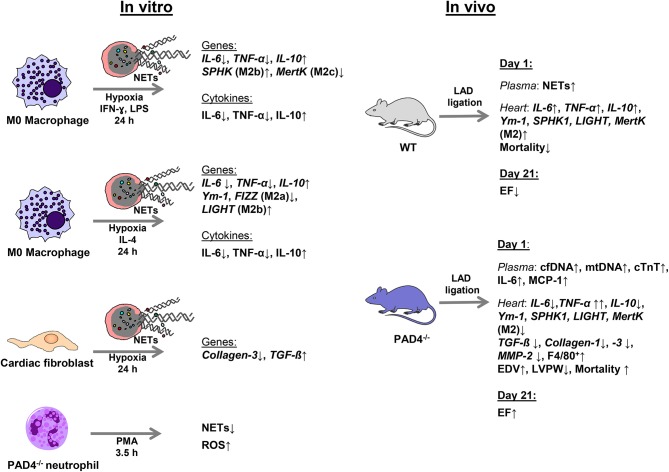
Schematic summary of the experimental design and main results.

As neutrophils persist in the infarcted myocardium for at least 7 days (45) and myocardial macrophages display high plasticity, changing their phenotype from M1 at day 1 to M2 at day 7 (11), NETs potentially modulate *in vivo* macrophage phenotypes. In accordance with the recent study published by Guimãraes-Costa et al. (46), here we found that NETs drive *in vitro* macrophage polarization toward an anti-inflammatory M2 phenotype under both M1- and M2a-polarizing culture conditions. Under M1-polarizing culture conditions, NETs were found to inhibit IL-6 and TNF-α expression and to upregulate IL-10 expression in macrophages. Similarly, NETs diminished markers of M2a macrophages in the presence of IL-4, again favoring IL-10 expression. In line with previously reported data (47), we confirm M2 polarization and an increase in IL-10 expression in a TLR-9-dependent manner. In turn, NETs-mediated IL-6 regulation under M1-polarizing conditions seemed to depend on other NETs-associated proteins. The main results of this study are summarized in Figure 8.

To further elaborate NETs effects on acute inflammation and cardiac healing, we used an acute MI model, which has already been reported to provoke cardiac neutrophil recruitment and strong release of inflammatory mediators (48). Histone citrullination by PAD4 is known to be necessary for chromatin decondensation during NETosis (49), and PAD4 deficiency was reported to result in the inability of neutrophils to release NETs (34, 50). Therefore, PAD4 deficiency should be accompanied by reduced levels of circulating nucleosomes/cfDNA. This assumption was initially supported by Savchenko et al. who found that plasma nucleosome generation in PAD4^−/−^ mice was lower than in WT mice in a 24 h myocardial reperfusion model (4). Having shown that PAD4^−/−^ mice display better recovery after MI, the authors concluded that NETs exacerbate ischemia/reperfusion injury. However, we show here for the first time, that PAD4^−/−^ mice subjected to permanent LAD ligation display significantly higher levels of circulating cfDNA and nucleosomes at day 1 post-MI when compared to WT controls. Thus, circulating nucleosomes/cfDNA do not generally reflect NET release by activated neutrophils and these results strongly contradict impaired cfDNA release in PAD4-deficient mice (4, 43) under our experimental conditions.

In our view, the reasons for exacerbated acute inflammatory response post-MI in PAD4^−/−^ mice seem to be multifaceted. As PAD4^−/−^ neutrophils were unable to form NETs *ex vivo*, the cfDNA increase found in PAD4^−/−^ mice is likely to be rather based on massive tissue injury as evidenced by increased plasma levels of cardiac troponin and mtDNA. In line with our assumption, plasma cfDNA isolated from PAD4^−/−^ mice at day 1 post-MI was highly pro-inflammatory, and strongly increased IL-6 expression in M1 macrophages. In this regard, TLR-9 activation by mtDNA was shown to provoke systemic inflammation (51), which is supported by the overwhelming pro-inflammatory response and increased mortality in PAD4^−/−^ mice in the acute phase post-MI.

In addition and in contrast to the data reported by Martinod et al. (52), PAD4^−/−^ neutrophils were found to produce higher ROS amounts than WT cells upon *in vitro* stimulation with PMA and ionomycin, respectively, although they did not release NETs under these conditions. NADPH oxidase inhibition did not abolish ROS production completely, probably due to enhanced production of mitochondrial ROS (53). In line with the findings recently reported (54), we found that PAD4 inhibition in WT neutrophils significantly increases PMA-triggered intracellular ROS production. These results suggest that NADPH oxidase becomes negatively regulated by active PAD4. Therefore, increased inflammation and cardiac injury in PAD4^−/−^ mice at day 1 post-MI is likely to be due in part to enhanced ROS release by high number of tissue-infiltrated leukocytes. However, here we failed to quantify lipid peroxidation in infarcted hearts, although no differences were found in plasma samples (unpublished results of our group).

Lastly, due to NETs deficiency in PAD4^−/−^ mice, anti-inflammatory mechanisms might be delayed, and impaired cardiac function and heart failure reported in neutrophil-depleted mice subjected to MI (55) may be attributed to compromised NETs release. Here, we found increased cardiac expression of *TNF-*α in infarcted hearts of PAD4^−/−^ mice, although *IL-6* expression was significantly diminished. In this respect, we found that NETs upregulate *IL-6* expression in the cardiac muscle cell line HL-1 (data not shown). Thus, NETs deficiency might be associated with impaired IL-6 production in cardiomyocytes. Because of the pleiotropic nature of this cytokine, it is not clear if reduced IL-6 expression in PAD4^−/−^ hearts promotes cardiac repair or aggravates inflammation. However, we recently reported that IL-6 drives polarization toward the M2b phenotype and a strong increase in IL-10 expression (56). Based on these previous results, reduced cardiac IL-6 expression in PAD4^−/−^ mice might explain reduced cardiac expression of M2/M2b macrophage markers. In a previous study, *IL-10* expression by cardiac macrophages and fibroblasts has been evidenced (57). As we did not find *IL-10* expression in cardiac fibroblasts isolated from WT hearts, neither in the absence nor in the presence of NETs (unpublished data), we assume that diminished cardiac *IL-10* expression in PAD4^−/−^ mice at day 1 after MI is indeed due to a reduced number of macrophages expressing M2b markers. Very recently, increased infarct size was reported after PAD4 inhibition with Cl-amidine and permanent ligation in mice. The authors concluded that the pathological process of the permanent ligation model is mainly affected by hypoxia and inflammatory response and speculated that cardiac repair requires a proper number of NETs (58). Accordingly, we also found slightly increased infarct sizes in PAD4^−/−^ mice at day 1 post-MI. This is probably also in part due to increased *MMP-2* expression found in PAD4^−/−^ mice, which may contribute to enhanced degradation of the extracellular matrix and enhanced infarct expansion (59). Increased thinning of the LVPW and higher EDV further indicate increased ventricular dilatation. However, we did not observe a significant reduction in LVEF at day 1, probably due to the low number of animals included.

One unexpected finding of this study was that all PAD4^−/−^ mice survived and displayed improved cardiac function in the post-acute phase (>day 1). In line with the data reported by Hemmers et al. (50), PAD4^−/−^ mice showed decreased weight loss already at day 3 post-MI. In addition, thicker LVPW at day 3 post-MI and significantly increased LVEF at day 21 were found when compared to infarcted hearts from WT mice. As PAD4^−/−^ mice did not upregulate cardiac *TGF-ß* expression at day 1 post-MI, consequently, expression levels of *collagen-1, collagen-3*, and *MMP-2* remained reduced when compared to the expression found in WT hearts. Indeed, here, NETs were found to upregulate *TGF-ß* expression in cardiac fibroblasts *in vitro*, which triggers the expression of collagens and MMP-2. However, no upregulation of *collagens* and *MMP-2* has been observed *in vitro*, suggesting a timely delayed regulation. Thus, NETs deficiency accompanied by reduced cardiac *TGF-ß* expression might result in moderate collagen deposition, thus increasing ventricular compliance.

However, to prove this assumption, the expression of collagens and MMPs should be monitored over time and the impact on cardiac fibrosis should be evaluated more deeply. In this context, it has already been demonstrated that old PAD4^−/−^ mice display better LVEF when compared to WT mice, which has been explained by reduced inflammation-related fibrosis. The authors also suggested that NETs drive fibrosis as *DNase I* infusion delayed the fibrotic process (60). As we demonstrate here that NETs exert anti-inflammatory properties and mice lacking functional PAD4 suffer from strong inflammation and increased mortality in the acute phase of MI, we hypothesize that improved cardiac recovery in the post-acute phase in PAD4^−/−^mice is largely based on compensatory mechanisms which cannot simply be explained by the lack of NETs. In line with this, at least two previous studies reported increased IL-10 levels in PAD4^−/−^ mice and concluded that the protective effect of PAD4 deficiency may be related to increased IL-10 production (43, 52). The cellular source of IL-10 has not been evaluated, but it was suggested that it might derive from non-NETing neutrophils (52). However, we cannot support this assumption as cardiac IL-10 levels at day 1 post-MI, e.g., when the number of cardiac neutrophils is proposed to be very high (45), were found to be significantly lower than in WT mice. Whether IL-10 becomes upregulated at later times in PAD4^−/−^ mice requires further study. Apart from its role in histone hypercitrullination, PAD4 is involved in gene regulation and PAD4 inhibitors have already been shown to increase Th_2_ responses (61) and to block the maturation of dendritic cells (62). In view of these multifaceted roles, it can be expected that PAD4 deficiency influences many immune cells and inflammatory pathways yielding in improved cardiac healing and ameliorated adverse remodeling. Importantly, in this pilot study we were not able to study the molecular mechanisms underlying exacerbated acute inflammation in PAD4^−/−^ mice in more detail, representing a major limitation of this study. Additional analyses are mandatory to explore the effects of NETs deficiency on cardiac macrophage polarization and inflammatory pathways in PAD4^−/−^ mice.

Altogether, our data suggest that the impaired cardiac upregulation of M2 genes in NETs-deficient mice favors cardiac inflammation in the acute phase after MI. Besides, dysregulated ROS production in PAD4^−/−^ neutrophils may contribute to increased tissue damage and subsequent cfDNA release in the circulation. We further provide evidence for the pro-inflammatory action of cfDNA. However, improved cardiac regeneration in PAD4^−/−^ mice has been observed. Studies using PAD4^−/−^ mice, which aim to assess NETs effects in acute inflammation, should be carefully reviewed as PAD4 deficiency consequentially seems to engage many compensatory mechanisms. Further studies, e.g., based on *DNase I* infusion, should be conducted to elaborate the role of NETs for cardiac healing and remodeling post-MI in more detail.

## Data Availability Statement

All datasets generated for this study are included in the manuscript/Supplementary Files.

## Ethics Statement

All animal studies were reviewed and approved by the local animal care committee (Bezirksregierung Köln, Germany, No.84-02.04.2014.A234) and complied with the national guidelines for the care and use of laboratory animals.

## Author Contributions

AP-G conceived and supervised the study and wrote the manuscript. KE performed surgeries and echocardiographic analyses and interpreted the results. LG performed echocardiographic analyses and *in vitro* experiments with neutrophils. LG, CW, TL, HZ, UK, and AC performed experiments and analyzed the results. MM and TW provided support with mouse surgeries and echocardiography. All authors participated in data analysis, critically reviewed the manuscript, and gave final approval.

### Conflict of Interest

The authors declare that the research was conducted in the absence of any commercial or financial relationships that could be construed as a potential conflict of interest.
