# Supplementary figures and images for: Compromised Anti-inflammatory Action of Neutrophil Extracellular Traps in PAD4-Deficient Mice Contributes to Aggravated Acute Inflammation After Myocardial Infarction
Source: Front Immunol. 2019 Oct 1;10:2313. doi: 10.3389/fimmu.2019.02313 (PMC6779806; doi:10.3389/fimmu.2019.02313)

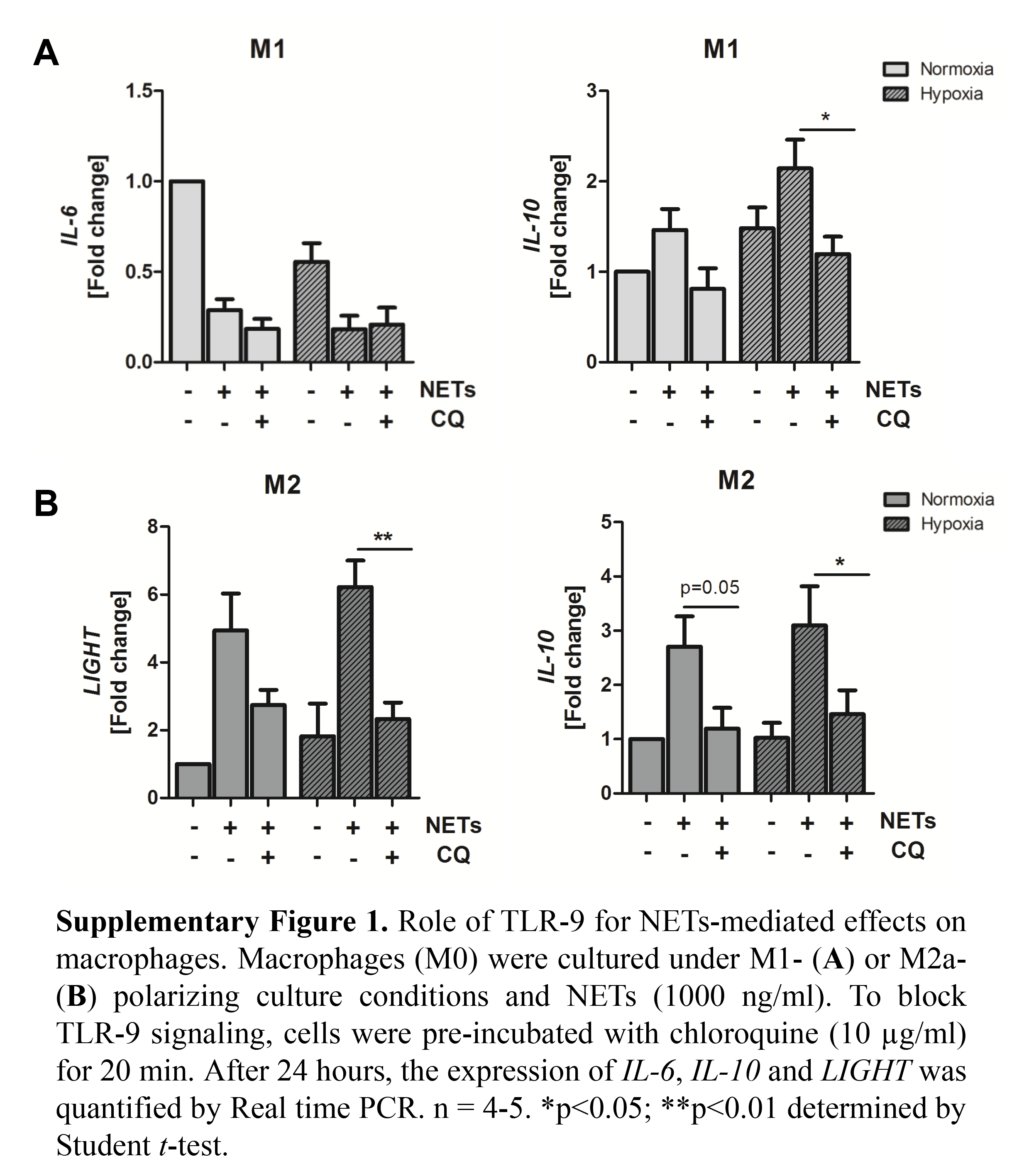

Supplement: Supplementary file 1 [file Image_1.TIF]
